# Chimpanzees monopolize and children take turns in a limited resource problem

**DOI:** 10.1038/s41598-019-44096-4

**Published:** 2019-05-20

**Authors:** Hagen Knofe, Jan Engelmann, Michael Tomasello, Esther Herrmann

**Affiliations:** 1 Minerva Research Group on the Origins of Human Self-Regulation, Max Planck Institute for Evolutionary Anthropology, Deutscher Platz 6, 04103 Leipzig, Germany; 2 Department of Psychology, Yale University, New Haven, Connecticut 06520-8205 USA; 3 Department of Developmental Psychology, Georg-Elias Müller Institute of Psychology, University of Göttingen, 37073 Göttingen, Germany; 4 Department of Psychology and Neuroscience, 417 Chapel Drive, Duke University, Durham, NC 27708 USA

**Keywords:** Evolution, Anthropology, Human behaviour

## Abstract

Competition over scarce resources is common across the animal kingdom. Here we investigate the strategies of chimpanzees and children in a limited resource problem. Both species were presented with a tug-of-war apparatus in which each individual in a dyad received a tool to access a reward, but tools could not be used simultaneously. We assessed the equality of tool use as well as the frequency of turn taking. Both species managed to overcome this conflict of interest but used different strategies to do so. While there was substantial variation in chimpanzee behaviour, monopolization was the common course of action: tool use was asymmetric with individual chimpanzees monopolizing the resource. In children, turn-taking emerged as the dominant strategy: tool use was symmetric and children alternated access to the tool at a high rate. These results suggest that while both species possess strategies for solving limited resource problems, humans might have evolved species unique motivations and socio-cognitive skills for dealing with such conflicts of interest.

## Introduction

By 2025, according to estimates by the United Nations, as much as two-thirds of the world population could be affected by water scarcity. When key resources, such as water, are finite, individual parties often compete for access. Competition for scarce resources not only emerges on a global level over resources such as water, food, or soil, but also in smaller-scale dyadic situations when two individuals face a conflict of interest over a given resource (e.g. food, mates, or special tools). Competition for limited resources is widespread in the animal kingdom: think, for example, of two hermit crabs who want to use the same seashell as a shelter or two penguins competing for the optimal breeding spot. One of our closest living relatives, chimpanzees, also routinely face such dilemmas, for example when two chimpanzees both want access to the same particular hammer tool in order to engage in nut-cracking.

Different strategies can be employed in limited resource problems. One such strategy is monopolization by which one party gains exclusive access to the resource. Monopolization is characterized by a highly asymmetric output: one individual can benefit from the resource while the majority remains empty handed. A second strategy is turn-taking. When turn-taking, individuals alternate who has access to the resource. Turn-taking is characterized by low levels or even an absence of asymmetric output: it is mutually profitable in the sense that all relevant individuals get a share of the resources. Between monopolisation and turn-taking is a continuum of strategies ranging from one extreme to the other.

Here, we are interested in the strategies young children and chimpanzees use in limited resource problems. Comparing children’s and chimpanzees’ strategies allows us to draw inferences about the evolution of these. There are a limited number of studies investigating young children’s behaviour in the context of access to scarce resources. One class of studies is observational in nature and prototypically involves observations of how two children regulate access to a desired resource, such as an attractive toy. This early research revealed that in such situations individual children often first monopolize the resource, but then after a while either spontaneously or as a result of solicitation agree to share^1–3^. While these results provide important insights into children’s treatment of limited resources, the inferences we can draw based on these studies regarding young children’s strategies in resource competition situations are limited. One reason for this is that the tasks presented to children were not clearly competitive in nature. In the study by Putallaz and Sheppard^2^, for example, children could also manipulate the toys in a cooperative manner (e.g. playing the computer game together). A second class of studies uses experimental protocols to investigate children’s strategies in limited resource problems. Madsen and colleagues designed a model tug-of-war situation^4–7^ in which dyads engaged in a marble pull game that involved a clear conflict of interest: one child had to refrain from pulling so that the other child could access the marble. If both children pulled at the same time, the marble fell into an inaccessible hole. While results show that children at least sometimes used turn-taking strategies, the authors’ main interest was whether children were able to maintain cooperation (and successfully retrieve the marble) in the face of opposing preferences, rather than in their strategies for doing so. Additional work by Zeidler and colleagues^8^ included a cross-cultural investigation of children’s behaviour in a similar limited resource problem.

There is no previous study on chimpanzees’ (*Pan troglodytes*) strategies in a limited resource context. However, a recent study by Melis, Grocke, Kalbitz, and Tomasello^9^ investigated whether human children and chimpanzees can develop turn-taking strategies in order to solve a recurrent cooperative dilemma. Using a set-up similar to the one used by Madsen^10^, individuals had to collaborate in order to produce a resource that could only be accessed by one of the two collaborative partners. From age 5 onwards, human children managed to maintain cooperation in this set-up by taking turns, that is, by alternating who would be the recipient of the reward. Chimpanzees, on the other hand, very rarely engaged in turn-taking and as a consequence the level of collaboration declined over time. However, the main focus of this study was whether cooperation could be maintained in the face of unequal rewards, and not on the strategies of chimpanzees and children in limited resource problems. One possibility is that having to actively collaborate on a task might have masked any turn-taking strategies or other strategies that chimpanzees might use in such a context. Furthermore, many of the limited resource problems chimpanzees face in their naturalistic environment seem to involve situations where there is a direct conflict of interest over a given resource, rather than a cooperative effort that results in unequal rewards.

**Figure 1:**
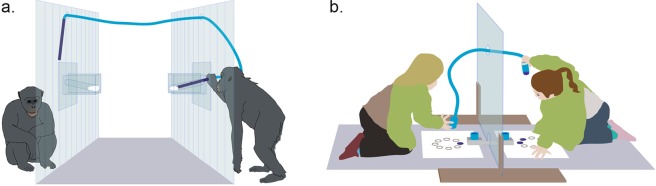
Schematic set-up for (**a**) chimpanzees and (**b**) children. (**a**) Two tools are tied to the same short rope, thereby preventing simultaneous access to the yogurt filled boxes. (**b**) Two stamps are tied to the same short rope, thereby preventing simultaneous access to the ink (which was needed to stamp the circles on the sheet). © Max Planck Institute for Evolutionary Anthropology.

Thus, in the current study, our aim was to investigate the strategies young children and chimpanzees use in competitive resource problems. We presented children and chimpanzees with a limited resource problem: restricted access to tools. Although each member of a dyad received their own tool, the trick was that the two tools were attached to one another by a short rope (see a. of Fig. 1 for an image of the chimpanzee set-up and b. of Fig. 1 for the children’s set-up). Thus, if both individuals pulled at the same time, no one could use the tool. In order for one individual to use their tool, the second individual had to let go of their tool. Our main variables of interest - allowing us to differentiate between monopolization and turn-taking strategies – were the inequality of tool use and the frequency of turn-taking in tool use.

## Methods

### Participants

Sixteen chimpanzees took part in the familiarization phase of this study. Since four chimpanzees did not pass all criteria during this phase (see the Supplementary Information [SI]), the final number of participants included twelve chimpanzees (7 females, 5 males, *M*_*age*_ = 16.08 years, age range: 6–36 years). With these individuals, we formed eighteen dyads. However, three of the eighteen dyads stopped manipulating the apparatus and were thus dropped leaving 15 dyads that participated in this study (for details see SI and Supplementary Table S1).

In addition, we tested eighty 5-year-old children in gender-matched dyads (*N* = 40 dyads, 40 girls, 40 boys, *M*_*age*_ = 5.08 years, age range = 4.85–5.35 years). Three child dyads were dropped from the study because they stopped participating. The children were mainly from from middle-class families and were tested in Kindergartens from a medium sized German city in the eastern part of the country.

The study complied with the European and World Associations of Zoos and Aquariums Ethical Guidelines and was approved by the joint ethical committee of the Max-Planck Institute for Evolutionary Anthropology and Leipzig Zoo. Chimpanzees were neither food- or water-deprived and could participate or refuse to participate in the study by their own choice. Child studies were carried out with the written informed consent of the parents and the verbal consent of the participants, and in accordance with all applicable laws and rules governing psychological research in Germany.

### Materials

All subjects were presented with an apparatus simulating a tug of war situation: a short rope connected two tools, thereby making simultaneous access to the rewards impossible. The chimpanzees competed over a stick needed to access a yogurt-baited box (see a. of Fig. 1). The children each needed to dip a stamp into an ink-filled tube to finish a pre-printed stamp-pattern (eight circles on a sheet of paper, see b. of Fig. 1). Both members of a dyad were able to see each other throughout the experiment.

### Procedure

All subjects were individually introduced to the apparatus and procedure during a familiarization phase. In the first two stages, chimpanzees needed to prove their tool-use skills and motivation (poking for yogurt with the stick-tool) as well as their understanding of the apparatus. To pass criterion in step three, chimpanzees needed to feed from both baited boxes (in this stage the door between both experimental rooms remained open). This last step encouraged subjects to take the perspective of the partner (not present in the familiarization phase) and granted an understanding of the mirrored set-up. The children experienced a single familiarization trial in which they learned how to use the stamp and the ink filled tubes and then had to demonstrate their stamping ability. At the end of the familiarization phase, all subjects had a solid understanding of the set up and its contingencies. For more information on familiarization please refer to the SI.

All subjects were tested in pairs. In the case of chimpanzees, the experimenter called the subjects’ names and simultaneously baited both boxes with two tablespoons of yoghurt. The trial started by simultaneously opening two sliding doors, allowing each chimpanzee to enter their respective test room. Afterwards, the experimenter left the testing area. After 10 minutes the yoghurt tubes were removed, ending the trial. All chimpanzee dyads received 10 test sessions, each consisting of a single trial (10 trials total).

Similar to the chimpanzees, children were first directed to their assigned side of the apparatus (which remained constant throughout the experiment). The experimenter explained the rules: the children were told to stay on their assigned side of the apparatus, use fresh ink for every new stamp and apply only one mark to each circle on their stamping sheet (there was a total of eight circles on each sheet and children received one sheet per trial). The experimenter then left the room and the trial started. A trial ended either after a maximum of 2.5 minutes, or after both children finished their stamping sheet. After each trial, the children left the room and the experimenter exchanged both stamps and stamping sheets (a new motif and stamping pattern was provided each trial). Each dyad received five trials in a single test session.

### Coding and analysis

All experiments were video-taped. Two main variables were coded from video: (A) the number of *individual tool-uses* and (B) the *number of turn-taking events*. Individual tool-uses (A) were defined as dipping the stick-tool into the box (for at least a third of its length) and pulling it out (chimpanzees) or stamping a postmark into an empty circle on the stamping-sheet (children). Thus, across species, a tool use was only coded after a full functional utilization of the tool. Based on the number of individual tool-uses (A) we calculated the *inequality of tool use*, which is the proportion of tool uses conducted by the subject who showed more tool use. This variable represented the proportional inequality of the number of individual tool-uses between two participants and allowed us to discern how much more one individual used the tool (if both individuals did not use it equally). The inequality of tool use could range from 0.5 (equal tool use) to 1 (complete monopolization by one individual). Turn-taking events (B) were defined as the number of changes in who is using the tool within the trials.

10% of the child data and 20% of the chimpanzee data were coded independently by research assistants who were unaware of the study design and research question. The inter-coder reliabilities (Spearman’s rank order correlations with exact p-values) were excellent for chimpanzees and children, respectively (individual tool uses: *r*_*s*_ [60] = 0.998, *p* < 0.001, *r*_*s*_ [40] = 1, *p* < 0.001; turn-taking events *r*_*s*_ [30] = 1, p < 0.001, *r*_*s*_ [20] = 1, p < 0.001).

Since we were interested in the strategies used by the two species to solve the limited resource problem, we calculated three Generalized Linear Mixed Models (GLMMs^11^). To test the effect of our single test predictor species, we always compared each full model with a null model (lacking the test predictor, keeping the control factors and random effect structure) using a likelihood ratio test (LRT). For all models, we ran several model diagnostics. These were all unproblematic.

In a first model, we looked at how the inequality varied between the two species on trial level. Then, in a second model, we investigated the level of inequality on a dyadic level since individuals could alternate in monopolizing the tool in each trial and thereby eventually end up with an equal outcome, too (see^8^). More specifically, we fitted two GLMMs with binomial error structure and logit link function to investigate how the inequality of tool use varied between the two species on a trial basis (addressing the level of inequality for every trial, GLMM 1) and on the dyadic level (addressing the overall inequality per dyad, across trials, GLMM 2). The response in both models was a two-columns matrix comprising number of tool uses by the individual who showed more tool use and the individual who showed less tool use. Such a response means that the model fits the proportion of tool uses that was conducted by the subject who showed more tool use. Both models controlled for the fixed effects friendship (two levels: ‘yes’; ‘’no’), sex/gender (three levels: ‘male’; ‘female’, ‘mixed’) kindergarten group (controlling for group affiliation in children; two levels: ‘yes’; ‘’no’; chimpanzees always coded as ‘yes’ since all chimpanzees were from the same group) and GLMM 1 for trial number. We included two random intercepts for the identities of the two subjects (grouped according to which individual showed more and which showed less tool use), a random intercept of dyad and in model 1 a random intercept of trial identity nested within dyad. To keep the type I error rate at the nominal level of 0.05 we included random slopes^12,13^ of trial number within the random effects of the two subjects and the dyad in model 1, but we did not include the correlations among the random slopes and intercepts.

In a third model with Poisson error structure and logit link function, we investigated the effect of species on the total number of turn-taking events per dyad across trials. We controlled for friendship, sex/gender and kindergarten group (fixed effects) and the effect of individuals on the response (two random effects, identity of each participant within a dyad). To account for inter-dyadic and/or inter-species differences in effort (number of tool uses and number of trials), potentially resulting in a different number of chances to take turns, offset terms were included. For detailed model descriptions and outputs see the SI. The data and R-code is available as part of the Supplementary Information.

## Results

**Figure 2:**
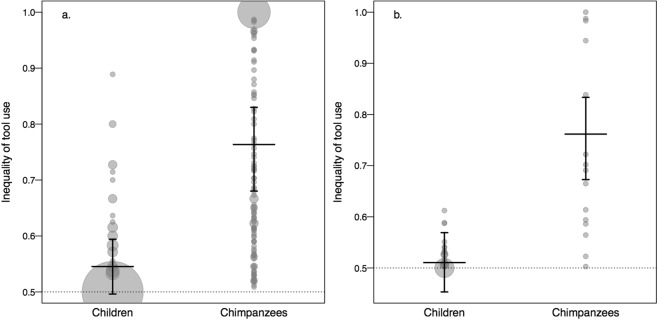
Estimated inequality of tool use for both species on trial by trial basis (**a**) and across trials (**b**). Estimates are depicted by a long horizontal line. Whiskers represent the confidence intervals. Inequality of tool use theoretically ranges from 0.5 (*equal tool use;* indicated by the dashed line), to 1 (*complete monopolization by one individual*). Grey circles show the full distribution of the data. Bigger circles represent more data points.

The first GLMM, that investigated each trial separately, found the proportional difference of tool use within a dyad more unequal in chimpanzees as compared to children (LRT: χ^2^ = 13.90, *df* = 1, *p* < 0.001; see a. of Fig. 2). On a dyadic level (GLMM 2), the proportional difference in tool use between the subjects was again more unequal in chimpanzees as compared to children (LRT: χ^2^ = 13.88, *df* = 1, *p* < 0.001; see b. of Fig. 2). None of the control variables (friendship, sex/gender, kindergarten group and trial number) significantly influenced the response. The chimpanzees’ tool-use differed significantly from equality, as the confidence intervals did not comprise the level of equality of 0.5 (*equal tool use*). Thus, one chimpanzee in a given dyad regularly received a greater share than the other chimpanzee, both within and across trials. However, chimpanzee dyads varied in their proportion of monopolization (see discussion). Children’s behaviour could not be distinguished from equal tool-use. Thus, children used their tools equally, given that the confidence intervals comprised the level of equality of 0.5 (*equal tool use*). This effect was enlarged across trials (see b. of Fig. 2).

**Figure 3:**
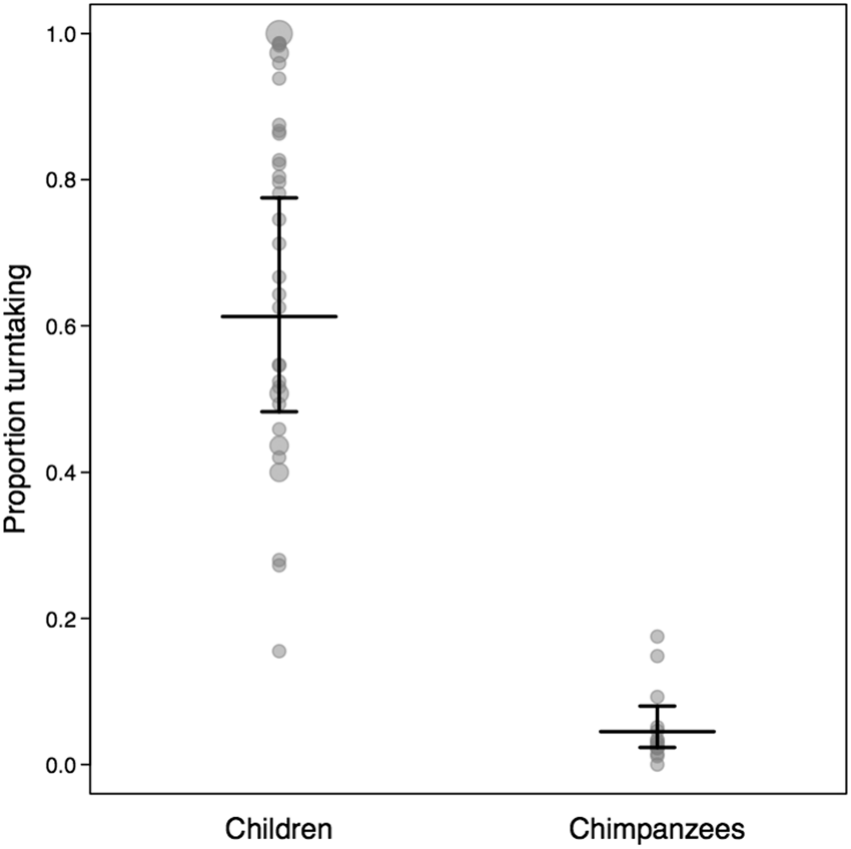
Estimated proportional turn-taking for chimpanzees and children. Estimates are depicted by a long horizontal line. Whiskers represent the confidence intervals. The proportional turn-taking scale theoretically ranges from 0 (*no turn-taking*) to 1 (*taking turns after each tool use*). Grey circles show the full distribution of the data. Bigger circles represent more data points.

The third model focused on the strategy of turn-taking. We found significantly more turn-taking in tool use in children than in the chimpanzees (LRT: χ^2^ = 67.58, df = 1, p < 0.001; see Fig. 3). Thus, the level of equality in tool-use reached by the tested children was achieved by taking turns within trials. The control variables had no significant influence on the response. For details on the model coefficients of all GLMMs see the Supplementary Table S2.

## Discussion

Here we investigated children’s and chimpanzees’ strategies in a limited resource problem. Both species were presented with a conflict of interest scenario: each member of a dyad needed a tool to access an attractive resource; crucially, however, the tool could only be accessed by one individual at a time. Children and chimpanzees showed markedly different strategies in this context. Children alternated access to the tool: they took turns such that each party in the dyad used the tool equally. Chimpanzees maximized individual output: in the majority of dyads, one individual monopolized access to the tool and thus received the larger share of the resource. The dominant strategy for children was thus turn-taking, and for chimpanzees, monopolization.

Our results with children confirm and extend previous work on children’s behaviour in competitive resource situations^2,8^. As measured both by the inequality of tool use as well as the frequency of turn-taking, children displayed a strong tendency to create equal output by alternating access to the tool. This is most evident from our analysis of children’s behaviour across trials. While some dyads showed an asymmetry in tool use within individual trials, this asymmetry was erased or at least minimized across trials. This suggests that although some children monopolized access to the tool in individual trials, who monopolized the tool alternated across trials, resulting in turn-taking behaviour. In line with this result, we also found that children showed a high rate of passing the tool back and forth. It is important to note, however, that the frequency of turn-taking alone does not license any inferences regarding symmetry or asymmetry of tool use. Even a single turn-taking event can generate a symmetric output. From a broader perspective, turn-taking can be classed as a special instance of reciprocal interaction. The fact that children in this study showed turn-taking is in line with recent work showing that young children’s reciprocal behaviour develops in middle childhood^14–16^.

In contrast to the children’s turn-taking, we found that chimpanzees mostly opted for a monopolization strategy. Within trials, output was asymmetric with monopolization emerging as the most frequent outcome. Furthermore, unlike the children, chimpanzees did not reduce output asymmetry across trials. Specifically, the fitted inequality of tool use both within and across trials was 76%, meaning that one chimpanzee accessed about three-quarters of the resource, leaving one-quarter to the partner. Importantly, however, we observed substantial variation in chimpanzee behaviour. Five chimpanzee dyads showed a nearly symmetric output across trials (between 50% and 60% inequality of tool use). The three dyads who had the most equal outcomes (Fifi-Trudi, Jahaga-Fifi, Riet-Tai) were all friends (see SI). The most equal dyad, Fifi-Trudi, had an inequality of tool use score of 0.5, meaning that output was perfectly balanced.

Dyadic cooperative relationships are based on an exchange of goods and services that is satisfactory to the involved parties. Previous work has shown that chimpanzees form long-term non-kin cooperative relationships that can last up to several years and are based on mutual trust^17–19^. The mostly unequal distribution of rewards observed in this study raises the question of how chimpanzees can maintain such relationships in the face of reward monopolization. One possibility is that accounting in chimpanzee relationships is based on long-term mechanisms that can tolerate substantial short-term inequalities. Support for this view comes from studies showing that chimpanzees do not balance services within single interactions but rather over repeated encounters^20–22^ and generally do not seem to show short-term forms of reciprocity^23,24^. By this account, immediate turn-taking is considered a cognitively demanding strategy that is adapted for facilitating balanced cooperative interactions among relative strangers who interact rarely^25^. Chimpanzees, on the other hand, live in relatively small and stable social groups that are characterized by repeated interactions rather than one-shot encounters. In chimpanzee social ecology, more long-term balancing mechanisms such as attitudinal reciprocity (also sometimes called emotional bookkeeping)^26,27^ might guarantee the stability of cooperative relationships. In line with this argument, Zeidler and colleagues^8^ show that also in humans, short-term turn-taking seems to be a more prevalent strategy in children from large-scale societies compared to children who grow up in small, close-knit communities.

One limitation of the current study is that our sample consisted exclusively of so-called WEIRD (Western, Educated, Industrialized, Rich, Democratic) children. Taking turns in response to limited resource problems might be a strategy that comes readily to the Western mind, but is absent in other cultures, thereby limiting the inferences we can draw from the current comparison of Western children’s and chimpanzees’ strategies. However, Zeidler and colleagues^8^ found that while children from two different Kenyan cultures (Kikuyu, Samburu) showed a very high likelihood of monopolizing a resource on the first of two trials, half of the dyads in both cultures then alternated who would monopolize the resource on the second trial, resulting in turn-taking behaviour across trials. In addition, Silk and House^15^ report that over ontogeny, similar patterns of reciprocity develop across diverse societies. Thus, although more cross-cultural evidence regarding strategies in limited resource problems is needed, it would seem that alternating access to a resource on an equal basis is not a strategy that is exclusive to Western children. A second limitation of the current study is that we focused on a dyadic version of a competitive resource problem. In many real-world contexts, not only individuals but whole communities or even countries compete for access to scarce resources, making turn-taking a practically and cognitively more demanding strategy. That is to say, strategies that can successfully solve dyadic resource competition might not map clearly onto solutions for multilateral conflicts. There is extensive literature in the political and economic sciences, for example on the topic of common-pool resources (e.g.^28^), exploring collective strategies for limited resource problems. This work has revealed that some of the strategies investigated here, turn-taking and monopolization, can also be scaled up and employed in multilateral settings. Nevertheless, future work should investigate how chimpanzees and children behave in competitive resource problems that involve a larger number of participants.

In the present studies, children and chimpanzees were exposed to a situation marked by conflict of interest. Child dyads overwhelmingly chose a mutually profitable strategy that allowed both children to finish their task. Chimpanzees showed substantial variation in their behaviour, but most dyads converged on an individually profitable course of action in which one individual consumed the majority of the resource. Both turn-taking and monopolization are possible solutions to limited resource problems. In a sense, children and chimpanzees both solved the current competitive resource problem: in neither species did mutual blocking appear as the dominant strategy. That is, there were no cases of one individual consistently preventing their partner from accessing the resource out of spite. The observation that children mostly opted for a turn-taking strategy and chimpanzees for a monopolization strategy might be grounded, as we have seen above, in their cooperative ecology. While chimpanzees mostly cooperate in the context of long-term cooperative relationships, humans have developed a novel cooperative tool kit – involving skills such as turn-taking – which allows them to create balanced interactions even in ephemeral interactions among strangers.

## Supplementary information

SI

Dataset 1
