# Supplementary material for: Chimpanzees monopolize and children take turns in a limited resource problem
Source: Sci Rep. 2019 May 20;9:7597. doi: 10.1038/s41598-019-44096-4 (PMC6527849; doi:10.1038/s41598-019-44096-4)
Supplement: Supplementary file 1 — SI [file 41598_2019_44096_MOESM1_ESM.pdf]

**Supplementary information (SI)**

# Chimpanzees monopolize and children take turns in a limited resource problem

Hagen Knofe, Jan Engelmann, Michael Tomasello and Esther Herrmann

*Appendix A: Participants and counterbalancing, design, drop out and repetition criteria, friendship data*

Chimpanzees: Sixteen chimpanzees took part in the familiarization phase of this study, see Table S1. From the twelve individuals who passed all familiarization criteria, we formed three same-sized test-groups (four individuals per group, see Table S1). Each contained one high ranking and one low ranking male and female. Note that just one male was available for group 3 because just one male lived in the housing group B. Within these groups every individual acted as a subject and was tested in dyads with every other group member (six dyads per group, eighteen in total; Note that three of the eighteen dyads stopped manipulating the apparatus and were dropped from the study). Table S1 depicts the 15 dyads that participated in this study.

All chimpanzee dyads received 10 test sessions, each consisting of one trial, 10 trials total. In the individual pretest, chimpanzees needed an average of three minutes to empty their yogurt-box. The maximum experimental trial length was ten minutes to provide sufficient time, in theory, for both individuals to finish their own portions. The interval between two test sessions within a dyad was restricted to a maximum of six days. If exceeded, the participants received one refresher trial of the third familiarization step (see Appendix B).

If both subjects did not touch the tool at least once, the trial was repeated within the same or the next test session. Three dyads (Robert-Riet, Riet-Lobo and Fifi-Alex) showed no interest in manipulating the apparatus once in four consecutive trials or twice in three consecutive trials and were excluded from the experiment. A total of 15 dyads finished the experiment and were included in the analyses. In rare cases, fighting within the chimpanzee groups before testing notably changed subjects' behavior (aggressive dominance displays, fear), which led to a loss of interest in the

apparatus. In these cases, trials had to be interrupted. They were repeated in the next session. On one occasion, subjects lost interest in the yogurt because it has previously been used as a vector for medicine. This trial was repeated in the next session.

To assess the influence of relationship quality, we derived friendship data from a questionnaire answered by the chimpanzee caregivers. The three most experienced caregivers of each housing group rated each chimpanzee test-dyad in terms of their relationship (scale: ‘very good’, ‘relatively good’, ‘neutral’, ‘relatively bad’, ‘very bad’). Dyads were rated as friends if their relationship was rated on average as relatively good or very good. All dyads with worse ratings were rated as non-friends.

Children: All child dyads received five trials in a single test session. We reduced the trial number to ensure children’s motivation remained as high as in the chimpanzee subjects. Children needed approximately 45 seconds to finish their stamping sheet in the individual pretest. Therefore, the trial length was set to 2.5 minutes to ensure both individuals had sufficient time to finish their task.

To assess friendship levels, before the experiment we asked participating dyads if they had ever played with each other before. Dyads were subsequently classified as friends if their answer was affirmative. In addition, half of the dyads were picked from the same kindergarten group while the other half of dyads was made up of children who had no or very limited previous interaction (i.e. they were recruited from different kindergarten groups).

**Table S1** *Chimpanzee participants and test dyads, N = 15*

| Subject | Sex | Age | Group | Successful familiarization | Test dyads | |
|---|---|---|---|---|---|---|
| | | | | | Subject 1 | Subject 2 |
| *Robert*[A] | m | 36 | 1 | Yes | | |
| Riet[A] | f | 34 | 1 | Yes | *Robert* | Tai |
| Tai[A] | f | 9 | 1 | Yes | *Robert* | *Lobo* |
| *Lobo*[A] | m | 7 | 1 | Yes | Riet | Tai |
| *Frodo*[A] | m | 18 | 2 | Yes | Tai | *Lobo* |
| *Kofi*[A] | m | 6 | 2 | Yes | *Frodo* | *Kofi* |
| Pia[A] | f | 12 | 2 | Yes | *Frodo* | Pia |
| Kara[A] | f | 6 | 2 | Yes | *Frodo* | Kara |
| Jahaga[B] | f | 19 | 3 | Yes | Pia | *Kofi* |
| Fifi[B] | f | 18 | 3 | Yes | Pia | Kara |
| Trudi[B] | f | 18 | 3 | Yes | *Kofi* | Kara |
| *Alex*[B] | m | 10 | 3 | Yes | Jahaga | Fifi |
| Natascha[A] | f | 31 | / | No | Jahaga | Trudi |
| Dorien[A] | f | 31 | / | No | Jahaga | *Alex* |
| Ulla[A] | f | 34 | / | No | Fifi | Trudi |
| Corrie[A] | f | 35 | / | No | Trudi | *Alex* |

*Note.* Superscripted A and B indicates the housing group of the individuals; f = female; m = male (in *italic*); age in years at the beginning of the study; group numbers represent the experimental subgroups counterbalanced for sex and rank (see Appendix C)

*Appendix B: Familiarization phase.*

Chimpanzees: Individually, all chimpanzees had to successfully complete three steps of a familiarization phase before they proceeded to the test phase. In the first step, the subjects had access to just one room with a single yogurt box. In order to access the bait, they needed to pull out the stick already placed inside the box and use it for poking. Step two resembled step one, but the stick was tied to the rope, introducing the tug of war-principle. Step three resembled the experimental set up: Two rooms were each provided with a baited box and the connected stick-tools. But in contrast to the experiment, the door between both experimental rooms remained open to test the chimpanzees' understanding of the mirrored setup. The subjects had to feed from the reward box in each room. In each of the three familiarization steps, the chimpanzees had to gain access to and feed from the provided food in three consecutive trials to meet criterion. Then they moved on to the next step. In cases where individuals failed, they repeated the former, easier step. The subjects switched the room they started in after every session to avoid a room preference in other studies (starting position changed from left to right side).

A total of twelve chimpanzees (75%) passed all criteria of the familiarization phase. Four individuals (25%) showed no or too little interest in the apparatus and were excluded from further testing (see Table S1). Ten individuals succeeded in consecutively passing all criteria whereas two chimpanzees needed one back shift to the former condition (Kofi repeated step one and Robert step two).

Human children: Individually, the participants were presented with a single familiarization trial. The setup was the same as in the experimental phase but without a partner (each side provided with a stamping sheet, an ink-tube and a rope-connected-stamp). First, the individuals forming the dyad were briefly introduced to

each other outside the experimental room. Then one of the children was assigned to one side of the apparatus, brought into the experimental room and taught how to stamp their sheet by an experimenter. Together, experimenter and subject practiced the technique. Here, all subjects learnt that they always need fresh ink for every stamp and are supposed to mark in each circle printed on the sheet. These two rules were repeated before every trial in the experiment. After one subject was familiar with the apparatus and the stamping technique, she left the room and the experimenter repeated the procedure with the second child.

*Appendix C: Analyses and results.*

For coding, the computer program INTERACT (Lab Suite Version 2011, 9.6.4.375) was used. For the analysis, the trial time of the chimpanzee experiment had to be reduced from 10 minutes to 9min 50s because of a video recording mistake.

Since we were interested in the strategies used by the two species to solve the limited resource problem, we fitted three Generalized Linear Mixed Models (GLMMs, [1]) fitted in R, version 3.4.0 [2] using the functions 'glmer' of the R-package lme4 [3].

First, we fitted two GLMMs with binomial error structure and logit link function to investigate how the equality of tool use varied between the two species on a trial basis (addressing the level of inequality for each and every trial, GLMM 1) and on the dyadic level (addressing the overall inequality per dyad, across trials GLMM 2), because, potentially, the partners could alternate in monopolizing the tool and end up with an equal outcome. Therefore, we collapsed the equality of tool use of all trials (for each dyad separately) to investigate the overall equality for each dyad in GLMM 2.

The response in both models was a two-columns matrix comprising number of tool uses by the individual who showed more tool use and the individual who showed less tool use. Such a response means that the model fits the proportion of tool uses that was conducted by the subject who showed more tool use. Effectively this means to model the inequality in tool use of the two individuals, whereby total equality means this proportion being .5, and complete monopolization (i.e., maximum inequality) it being 1. Model 1 and 2 we fitted with binomial error structure and logit link function. The key test predictor in both models was 'species' which we included

as a fixed effect. Both models further controlled for the fixed effects of friendship, sex/gender, and same kindergarten group (two levels: 'yes' or 'no'; always 'yes' for chimpanzees; 50% of the children were recruited from the same and 50% from a different group within the same institution) and model 1 also for trial number. We included two random intercepts for the identities of the two subjects (grouped according to which individual showed more and which showed less tool use; when both subjects showed the same number of tool uses these were randomly assigned to the two random effects), a random intercept of dyad and in model 1 a random intercept of trial identity nested within dyad. To keep the type I error rate at the nominal level of .05 we included random slopes [4,5] of trial number within the random effects of the two subjects and the dyad in model 1, but we did not include the correlations among the random slopes and intercepts.

Model 3 tested for species differences in the total number of turn taking events (response) per dyad. The model was a GLMM, fitted with a Poisson error distribution and log link function [6]. To account for number of tool uses differing between dyads (limiting the possible number of turns taken) we included the number of possible turn taking events (total number of tool uses – 1; log transformed) as an offset term into the model [6]. With regard to the fixed effects this model was identical to model 2, and we again considered species the key test predictor. As random intercepts, we included the identity of the two individuals per dyad (no random slope was identifiable since none of the fixed effects varied sufficiently within the two random effects; and a random intercept of dyad was not needed since each dyad provided only one value for the response).

To test the effect of our single test predictor, we always compared each full model with a corresponding null model (lacking the test predictor 'species' but

comprising the same control factors and random effect structure) using a likelihood ratio test [7]. For all models, we assessed several model diagnostics: Model stability was assessed by comparing the estimates of a given full model with the estimates obtained from models with the levels of the random effects excluded one at a time. This revealed no heavily influential levels of random effects to exist, particularly not for the effect of species. The assumption of normally distributed random effect components was met (assessed by visual inspection of the distributions of the conditional modes of the random effects). None of the three models was overdispersed (dispersion parameters; Model 1: .374; Model 2: .147; Model 3: .286). We checked for collinearity via Variance Inflation Factors (R package car; [8]) derived for a standard linear model lacking the random effects. This revealed no collinearity issues to exist (maximum squared Generalized VIF = 2.71 (after taking them to the power of $1/(2\times df)$, with df being the degrees of freedom for the respective term) [9]. The sample sizes for the three models were 350 observations of inequality of tool use within trials of 15 chimpanzee and 40 human children modeled as 88 individuals that showed less tool use and 87 individuals that showed more tool use grouped in 55 dyads (model 1); a total of 55 observations made for 50 individuals that showed less tool use, 49 individuals that showed more tool use, grouped in 55 dyads (model 2); and a total of 55 dyadic turn taking measures taken for 49 and 48 individuals grouped in 55 dyads (model 3). For a summary of the model coefficients of all three GLMMs see Table S2. The R code of each model is indicated below Table S2.

Table S2

*Model coefficients of main effect species and controlling effects on the respective response (GLMM 1-3)*

| GLMM | Term | *b* | *SE* | 95 % CI | | $\chi 2$ | *DF* | *p* |
|---|---|---|---|---|---|---|---|---|
| | | | | Lower | Upper | | | |
| 1 | Intercept | .085 | .156 | -.225 | .409 | NA | NA | NA |
| | Species_chimp | .989 | .252 | .488 | 1.456 | 13.899 | 1 | < .001 |
| | Sex_male | .141 | .164 | -.173 | .445 | 2.387 | 2 | .303 |
| | Sex_mixed | .315 | .209 | -.053 | .658 | NA | NA | NA |
| | Friends_ yes | -.1 | .190 | -.492 | .252 | .276 | 1 | .599 |
| | Kindergarten_ group_same | .052 | .229 | -.372 | .503 | .051 | 1 | .822 |
| | z.Trial_no | .085 | .064 | -.032 | .218 | 1.616 | 1 | .204 |
| 2 | Intercept | -.069 | .194 | -.457 | .325 | NA | NA | NA |
| | Species_chimp | 1.120 | .283 | .560 | 1.666 | 13.882 | 1 | < .001 |
| | Sex_male | .274 | .204 | -.151 | .694 | 1.931 | 2 | .381 |
| | Sex_mixed | .032 | .214 | -.277 | .324 | NA | NA | NA |
| | Friends_ yes | .052 | .244 | -.449 | .514 | .044 | 1 | .833 |
| | Kindergarten_ group_same | -.054 | .292 | -.647 | .514 | .034 | 1 | .854 |
| 3 | Intercept | -.294 | .136 | -.672 | .065 | NA | NA | NA |
| | Species_chimp | -2.610 | .228 | -3.204 | -2.000 | 67.576 | 1 | < .001 |
| | Sex_male | -.037 | .143 | -.415 | .350 | 0.509 | 2 | .775 |
| | Sex_mixed | -.147 | .210 | -.757 | .472 | NA | NA | NA |
| | Friends_ yes | .024 | .181 | -.456 | .499 | 0.018 | 1 | .893 |
| | Kindergarten_ group_same | -.275 | .211 | -.842 | .316 | 1.612 | 1 | .204 |

*Note. b* = Estimate, *SE* = standard error; *CI* = Confidence interval; NA = not available: data for the Likelihood ratio tests ($\chi^2$) not meaningful for Intercepts and levels of the fixed effects ($\chi^2$ always represents the factor and not the depicted level of it)

R code of GLMM 1:

```
all.data=read.table(file="location of the Data in .txt format", header=T, sep="\t", fill=T)
subjects=as.matrix(all.data[, c("Left_subject_Es_view.", "Right_subject_Es_view.")])
resp=as.matrix(all.data[, c("Stamps_left_subject", "Stamps_right_subject")])
for(i in 1:nrow(all.data)){
if(length(unique(resp[i, ]))>1){
subjects[i, ]=subjects[i, ][order(resp[i, ])]}else{
subjects[i, ]=subjects[i, ][sample(1:2, 2, replace=F)] resp[i, ]=resp[i, ][order(resp[i, ])]}}
colnames(subjects)=c("subj.less", "subj.more")
colnames(resp)=c("less", "more")
resp=resp[, 2:1]
subjects=subjects[, 2:1]
test.data=data.frame(subjects, resp, all.data[, c("species","Sex", "Friends_played_before",
"Kindergarten_groups_of_the_subjects", "Trial_no")])
test.data$dyad=as.factor(apply(subjects, 1, function(x){paste(sort(x), collapse="@")}))
trial.id=as.factor(1:nrow(test.data))
test.data$z.Trial_no=as.vector(scale(test.data$Trial_no))
contr=glmerControl(optimizer="bobyqa", optCtrl=list(maxfun=100000))
full=glmer(resp~species+Sex+Friends_played_before+Kindergarten_groups_
of_the_subjects+z.Trial_no+(1+z.Trial_no||subj.less)+(1+z.Trial_no||subj.more)+(1+z.Trial_no||dyad
)+(1|trial.id), family=binomial, data=test.data, control=contr)
null=glmer(resp~Sex+Friends_played_before+Kindergarten_groups_of_the_subjects+z.Trial_
no+(1+z.Trial_no||subj.less)+(1+z.Trial_no||subj.more)+(1+z.Trial_no||dyad)+(1|trial.id),
family=binomial, data=test.data, control=contr)
c.tab(as.data.frame(anova(null, full, test="Chisq")), 3)
summary(full)$coefficients, 3))
c.tab(as.data.frame(drop1(full, test="Chisq")),3)
```

R code of GLMM 2:

```
vars=c("Left_subject_Es_view.", "Right_subject_Es_view.", "species", "Sex",
"Friends_played_before", "Kindergarten_groups_of_the_subjects")
dyadic.test.data=aggregate(all.data$Stamps_left_subject, all.data[, vars], sum)
colnames(dyadic.test.data)[ncol(dyadic.test.data)]="pokes.left.subject"
dyadic.test.data$pokes.right.subject=aggregate(all.data$Stamps_right_subject, all.data[,
vars], sum)$x
subjects=as.matrix(dyadic.test.data[, c("Left_subject_Es_view.", "Right_subject_Es_view.")])
resp=as.matrix(dyadic.test.data[, c("pokes.left.subject", "pokes.right.subject")])
for(i in 1:nrow(dyadic.test.data)){
if(length(unique(resp[i, ]))>1){
subjects[i, ]=subjects[i, ][order(resp[i, ])]}else{
subjects[i, ]=subjects[i, ][sample(1:2, 2, replace=F)]}
resp[i, ]=resp[i, ][order(resp[i, ])]}
colnames(subjects)=c("subj.less", "subj.more")
colnames(resp)=c("less", "more")
resp=resp[, 2:1]
subjects=subjects[, 2:1]
dyadic.test.data=data.frame(dyadic.test.data, subjects)
dyadic.test.data$dyad=as.factor(apply(subjects, 1, function(x){
paste(sort(x), collapse="@")}))
full=glmer(resp~species+Sex+Friends_played_before+Kindergarten_groups_of_the_subjects
+(1|subj.less)+(1|subj.more)+(1|dyad),
family=binomial, data=dyadic.test.data, control=contr)
null=glmer(resp~Sex+Friends_played_before+Kindergarten_groups_of_the_subjects+(1|subj
.less)+(1|subj.more)+(1|dyad),
```

```
family=binomial, data=dyadic.test.data, control=contr)
c.tab(as.data.frame(anova(null, full, test="Chisq")), 3)
summary(full)$coefficients, 3))
c.tab(as.data.frame(drop1(full, test="Chisq")),3)
```

R code of GLMM 3:

```
plot(table(all.data$Total_tatus_within_75max))
total.pokes=tapply(all.data$Stamps_total, all.data$Dyad_number, sum)
n.trials=tapply(all.data$Stamps_total, all.data$Dyad_number, length)
chances=total.pokes-n.trials
chances=chances[match(as.character(all.data$Dyad_number), names(chances))]
sum(as.character(all.data$Dyad_number)!=names(chances))
contr=glmerControl(optimizer="bobyqa")
contr$optCtrl=list(maxfun=10000)
dyad=apply(as.matrix(all.data[, c("Left_subject_Es_view.", "Right_subject_Es_view.")]), 1,
function(x){paste(sort(x), collapse="_")})
dyad=as.factor(dyad)
full=glmer(Total_tatus_within_75max~species+Sex+Friends_played_before+Kindergarten_gr
oups_of_the_subjects+offset(log(chances))+
(1|Left_subject_Es_view.)+(1|Right_subject_Es_view.), data=all.data, family=poisson, control=contr)
null=glmer(Total_tatus_within_75max~+Sex+Friends_played_before+Kindergarten_groups_
of_the_subjects+offset(log(chances))+
(1|Left_subject_Es_view.)+(1|Right_subject_Es_view.), data=all.data, family=poisson, control=contr)
c.tab(as.data.frame(anova(null, full, test="Chisq")), 3)
summary(full)$coefficients, 3))
c.tab(as.data.frame(drop1(full, test="Chisq")),3)
```

*Appendix D: Additional analyses and results.*

As suggested, we ran additional analyses, which included the interaction of species*friendship keeping the structure of the original models (as described in the manuscript and above). We also added species*trial number to Additional GLMM 1 since the equality of tool use might change differently with increasing trial number between chimpanzees and children. We always compared each full model with a null model (lacking the main test predictor species and its interactions keeping the control factors and random effect structure) using a likelihood ratio test (LRT). If the full null model comparison was significant the coefficients were investigated further using the function drop one of lme4. If the implemented interactions (including species) were non-significant, the effect of species was calculated using a reduced model (lacking the interactions from the full model but keeping the main and control factors and random effect structure) and comparing it to a new model (lacking the interactions from the full model and the factor species but keeping the main and control factors and random effect structure).

The additional models were significant when compared to the null model (Additional GLMM 1, LRT: $\chi^2 = 16.41$, $df = 3$, $p < .001$; Additional GLMM 2, LRT: $\chi^2 = 14.03$, $df = 2$, $p < .001$; Additional GLMM 3, LRT: $\chi^2 = 68.35$, $df = 2$, $p < .001$). The interactions of species with friendship (implemented in all additional GLMMs) or trial number (only in Additional GLMM 1) did not influence the response variables in any of the three models. For details on the model coefficients of all Additional GLMMs see the supplementary Table S3. The effect of species was calculated using reduced models (keeping the main and control factors and random effect structure but removing the insignificant interactions). Reduced model 1, that investigated each trial

separately, found the proportional difference of tool use within a dyad more unequal in chimpanzees as compared to children (LRT: $\chi^2$ = 7.10, *df* = 1, *p* = .008). On a dyadic level (Reduced model 2), the proportional difference in tool use between the subjects was again more unequal in chimpanzees as compared to children (LRT: $\chi^2$ = 13.88, *df* = 1, *p* < .001). Reduced model 3 found significantly more turn-taking in tool use in children than in the chimpanzees (LRT: $\chi^2$ = 67.58, *df* = 1, *p* < .001). None of the control variables (friendship, sex/gender, kindergarten group and trial number) significantly influenced the response in any model. For details on the model coefficients of all Reduced GLMMs see the supplementary Table S4.

Summarising, the additional analyses replicated the original results. The R code (full and null models) is indicated below Table S4. The code needed for defining model terms (e.g. “resp”) is identical with the R code of the original models (GLMM 1-3; R code indicated in Appendix C.)

Table S3

*Model coefficients  of the Additional GLMMs 1-3*

| GLMM | Term | *b* | *SE* | *χ2* | *DF* | *p* |
|---|---|---|---|---|---|---|
| Addl 1 | Intercept | -.014 | .172 | NA | NA | NA |
| | Species_chimp | 1.491 | .456 | NA | NA | NA |
| | Friends_ yes | .208 | .313 | NA | NA | NA |
| | z.Trial_no | -.006 | .101 | NA | NA | NA |
| | Kindergarten_ group_same | -.201 | .305 | .434 | 1 | .510 |
| | Sex_male | .157 | .167 | 1.609 | 2 | .447 |
| | Sex_mixed | .239 | .218 | NA | NA | NA |
| | Species_chimp:Friends_ yes | -.495 | .392 | 1.590 | 1 | .207 |
| | Species_chimp: z.Trial_no | .158 | .158. | 1.532 | 1 | .216 |
| Addl 2 | Intercept | -.095 | .206 | NA | NA | NA |
| | Species_chimp | 1.296 | .546 | NA | NA | NA |
| | Friends_ yes | .166 | .389 | NA | NA | NA |
| | Sex_male | .281 | .205 | 2.053 | 2 | .358 |
| | Sex_mixed | .014 | .220 | NA | NA | NA |
| | Kindergarten_ group_same | -.146 | .380 | .147 | 1 | .702 |
| | Species_chimp:Friends_ yes | -.188 | .498 | .143 | 1 | .705 |
| Addl 3 | Intercept | -3.147 | .364 | NA | NA | NA |
| | Species_human | 2.900 | .391 | NA | NA | NA |
| | Friends_ yes | .147 | .209 | NA | NA | NA |
| | Kindergarten_ group_same | -.122 | .264 | 0.214 | 1 | .644 |
| | Sex_male | -.054 | .141 | 0.379 | 2 | .827 |
| | Sex_mixed | -.111 | .204 | NA | NA | NA |
| | Species_human: Friends_ yes | -.311 | .342 | 0.782 | 1 | .377 |

*Note.* Addl = Additional, *b* = Estimate, *SE* = standard error; NA = not available: data for the Likelihood ratio tests ($\chi^2$) not meaningful for Intercepts and levels of the fixed effects ($\chi^2$ always represents the factor and not the depicted level of it); : = interaction between factors

Table S4

*Model coefficients of the Reduced models 1-3*

| GLMM | Term | *b* | *SE* | *χ2* | *DF* | *p* |
|---|---|---|---|---|---|---|
| Red 1 | Intercept | .948 | .390 | NA | NA | NA |
| | Species_human | -.977 | .356 | 7.104 | 1 | .008 |
| | Friends_ yes | -.194 | .284 | .460 | 1 | .497 |
| | z.Trial_no | -.080 | .066 | 1.393 | 1 | .238 |
| | Kindergarten_ group_same | .126 | .329 | .146 | 1 | .702 |
| | Sex_male | .123 | .233 | 3.481 | 2 | .175 |
| | Sex_mixed | -.578 | .335 | NA | NA | NA |
| Red 2 | Intercept | -.069 | .194 | NA | NA | NA |
| | Species_chimp | 1.120 | .283 | 13.882 | 1 | < .001 |
| | Sex_male | .274 | .204 | 1.931 | 2 | .381 |
| | Sex_mixed | .032 | .214 | NA | NA | NA |
| | Friends_ yes | .052 | .244 | .044 | 1 | .833 |
| | Kindergarten_ group_same | -.054 | .292 | .034 | 1 | .854 |
| Red 3 | Intercept | -.294 | .136 | NA | NA | NA |
| | Species_chimp | -2.610 | .228 | 67.576 | 1 | < .001 |
| | Sex_male | -.037 | .143 | 0.509 | 2 | .775 |
| | Sex_mixed | -.147 | .210 | NA | NA | NA |
| | Friends_ yes | .024 | .181 | 0.018 | 1 | .893 |
| | Kindergarten_ group_same | -.275 | .211 | 1.612 | 1 | .204 |

*Note.* Red = Reduced, *b* = Estimate, *SE* = standard error; NA = not available: data for the Likelihood ratio tests ($\chi^2$) not meaningful for Intercepts and levels of the fixed effects ($\chi^2$ always represents the factor and not the depicted level of it); : = interaction between factors

R code of Additional GLMM 1

```
trial.id=as.factor(1:nrow(all.data))
all.data$z.Trial_no=as.vector(scale(all.data$Trial_no))
full.i=glmer(resp~species*(Friends_played_before+z.Trial_no)+Kindergarten_groups_of_the_subjects+Sex+(1+z.Trial_no||subj.less)+(1+z.Trial_no||subj.more)+(1+z.Trial_no||dyad)+(1|trial.id), family=binomial, data=all.data, control=contr)
null.i=glmer(resp~Friends_played_before+z.Trial_no+Kindergarten_groups_of_the_subjects+Sex+(1+z.Trial_no||subj.less)+(1+z.Trial_no||subj.more)+(1+z.Trial_no||dyad)+(1|trial.id), family=binomial, data=test.data, control=contr)
```

R code of Additional GLMM 2

```
full.i=glmer(resp~species*Friends_played_before.+Sex+Kindergarten_groups_of_the_subjects+(1|subj.less)+(1|subj.more)+(1|dyad),family=binomial, data=dyadic.test.data, control=contr)
null.i=glmer(resp~Friends_played_before.+Sex+Kindergarten_groups_of_the_subjects+(1|subj.less)+(1|subj.more)+(1|dyad),family=binomial, data=dyadic.test.data, control=contr)
```

R code of Additional GLMM 3

```
full.i=glmer(Total_tatus_within_75max~species*Friends_played_before.+Kindergarten_groups_of_the_subjects+Sex+offset(log(chances))+(1|Left_subject_Es_view.)+(1|Right_subject_Es_view.), data=all.data, family=poisson, control=contr)
null.i=glmer(Total_tatus_within_75max~Friends_played_before.+Kindergarten_groups_of_the_subjects+Sex+offset(log(chances))+(1|Left_subject_Es_view.)+(1|Right_subject_Es_view.), data=all.data, family=poisson, control=contr)
```

## References

1 Baayen, R. H. *Analyzing Linguistic Data: A Practical Introduction to Statistics Using R*. (Cambridge University Press, 2008).

2 R: A language and environment for statistical computing. v. version 3.4.0 (R Foundation for Statistical Computing, Vienna, Austria, 2014).

3 lme4: Linear mixed-effects models using Eigen and S4. R package version 1.1-7 (2014).

4 Schielzeth, H. & Forstmeier, W. Conclusions beyond support: overconfident estimates in mixed models. *Behav Ecol* **20**, 416-420, doi:10.1093/beheco/arn145 (2009).

5 Barr, D. J., Levy, R., Scheepers, C. & Tily, H. J. Random effects structure for confirmatory hypothesis testing: Keep it maximal. *J Mem Lang* **68**, doi:10.1016/j.jml.2012.11.001 (2013).

6 McCullagh, P. & Nelder, J. A. *Generalized linear models*. (Chapman and Hall, 1989).

7 Dobson, A. J. *An Introduction to Generalized Linear Models*. 2nd edn, (Chapman & Hall/CRC, 2002).

8 Fox, J. & Weisberg, S. *An R companion to applied regression*. 2nd edn, (Sage, 2011).

9 Fox, J. & Monette, G. Generalized Collinearity Diagnostics. *Journal of the American Statistical Association* **87**, 178-183, doi:10.1080/01621459.1992.10475190 (1992).
